# Effective strategies for increasing the uptake of modern methods of family planning in South Asia: a systematic review and meta-analysis

**DOI:** 10.1186/s12905-023-02859-2

**Published:** 2024-01-03

**Authors:** Zahid Ali Memon, Tahmeena, Syeda Aleena Fazal, Sophie Reale, Rachael Spencer, Zulfiqar Bhutta, Hora Soltani

**Affiliations:** 1 Centre of Excellence in Women and Child Health, Aga Khan University, Karachi, Pakistan; 2 Health Research Institute, Sheffield Hallam University, Sheffield, UK; 3 College of Health, Wellbeing and Life Sciences, Sheffield Hallam University, Sheffield, UK; 4 Department of Nursing and Midwifery, Sheffield Hallam University, Sheffield, UK; 5 Institute for Global Health and Development, Aga Khan University, Karachi, Pakistan

**Keywords:** Systematic review, Meta-analysis, Family planning, Demand-side intervention, Supply-side intervention, Effectiveness, Contraception

## Abstract

**Background:**

Family planning (FP) interventions have improved the use of modern contraceptives, yet a high unmet need for contraception still exists in South Asia. This systematic review of existing research was conducted to identify effective FP interventions that led to an increase in the uptake of modern methods of contraception in South Asia.

**Methods:**

Five electronic databases were searched for relevant studies published between January 1st, 2000 and May 4, 2023. Experimental studies that reported data on the impact of FP interventions on modern contraceptive use among women of reproductive age (15–49 years) in the South Asian region were included. A random-effects Inverse Variance weighted model was employed to pool the adjusted odds ratio (OR) on modern contraceptive use and unmet need for contraception. In addition, we computed subgroup meta-estimates based on intervention type and the urban-rural divide.

**Results:**

Among 643 studies identified, 21 met the inclusion criteria. The overall pooled odds ratio for modern contraceptive use was significantly higher (OR 1.51; 95% CI 1.35–1.70; heterogeneity; I^2^ = 81%) for FP interventions with a significant reduction in unmet need for contraception (OR 0.86; 95% CI 0.78–0.94, I^2^ = 50%). The subgroup analysis revealed demand-generation (OR 1.61; 95% CI 1.32–1.96), health system integrated (OR 1.53; 95% CI 1.07–2.20), and franchised FP clinic interventions (OR 1.32; 95% CI 1.21–1.44) had promoted the modern contraceptive uptake. Further, FP interventions implemented in urban settings showed a higher increase in modern contraceptive use (OR 1.73; 95% CI 1.44–2.07) compared to rural settings (OR 1.46; 95% CI 1.28–1.66). Given the considerable heterogeneity observed across studies and the low degree of certainty indicated by the GRADE summary for the primary outcome, caution is advised when interpreting the results.

**Conclusion:**

The review collated experimentally evaluated FP interventions that increased modern contraception use and reduced the unmet need in South Asia. The demand generation interventions were the most effective in increasing the uptake of modern contraceptive methods. Furthermore, the urban environment provides a conducive environment for interventions to improve contraceptive usage. However, further studies should assess which aspects were most effective on attitudes towards contraception, selection of more effective methods, and contraceptive behaviors.

**Supplementary Information:**

The online version contains supplementary material available at 10.1186/s12905-023-02859-2.

## Introduction

Family planning (FP) is a key strategy to improve women’s reproductive health and reinforce women’s autonomy through informed choices about their sexual and reproductive needs [1]. By enabling couples to achieve their desired family size and promoting optimal birth intervals, FP interventions have reduced fertility rates and improved maternal and child health outcomes globally [2]. In addition, at a broader global level, FP can have significant implications in achieving universal goals of economic development, poverty reduction, and environmental sustainability [3].

Modern contraceptive use is an important indicator of reproductive health and FP program success. The optimal use of modern contraceptives can speculate approximately a 32% reduction in maternal deaths and avert 90% of unsafe abortion-related deaths globally [2, 4]. From 1990 to 2019, worldwide contraceptive use among women of reproductive age (15–49 years) has increased from 554 million to 922 million, with a decline in global fertility rates from 3.2 to 2.5 live births per woman [5]. Over the years, this surge in contraceptive use has been attributed to various FP interventions, establishing FP as an effective investment and feasible public health intervention [6].

Despite progress, there are 232 million women of reproductive age (15–49 years) in low-income and middle-income countries (LMICs) [7] who want to prevent their pregnancies but are not using any modern contraceptives [8, 9]. South Asia, in particular, has the second-highest burden of maternal mortality after Africa, indicating poor reproductive health. Even though the worldwide usage of modern contraceptives has increased over the last few decades, it remains low in the South Asian region at 42% compared to the global average of 49% [4]. Approximately 17% of women in South Asia still have an unmet need for FP services [10] and 9% are not using any modern methods, relying on less effective traditional methods that can lead to unintended pregnancies, unsafe abortions, and higher maternal and neonatal mortalities [11].

Country disaggregated data from South Asia highlight significant disparities in modern contraceptive use and fertility rates both across and within countries [12]. The frequently reported barriers to modern contraceptive use among Asian women include cultural norms, lack of method knowledge, fear of side effects, and religious restrictions [13, 14]. Nonetheless, noteworthy accomplishments have been observed in certain countries with regard to the decline in fertility rates by eliminating barriers to modern contraception and improving access to FP services. In Bangladesh, the effective implementation of FP interventions has resulted in a steady decline in fertility rates [15, 16]. Other studies have evidenced that India [17] Indonesia [18, 19] and Iran [20] have experienced accelerated contraceptive use owing to FP interventions and by educating females.

Within FP interventions, a number of different strategies can be employed to achieve the desired effect and outcome. A systematic review of 63 studies (conducted between 1994 and 2008 in LMICs) concluded that both demand-generation and supply-side interventions successfully increase knowledge, improve attitudes and beliefs, and facilitate effective communication around FP. This review further emphasized that FP program success has not been uniform in all locales and that no single solution fits all contexts. As such, tailoring complex FP interventions to improve women’s education and decision-making could help improve modern contraceptive use [21].

Furthermore, another review reported improved contraceptive use in LMICs by evaluating demand-side interventions and their cost-effectiveness [22]. Other subsequent reviews have demonstrated the efficiency of various standalone interventions in improving contraception use among adolescents and women of reproductive age (15–49 years) in LMICs, such as educational strategies to improve postpartum contraceptive use [23], social networking site strategies [24], and vouchers programs [25].

While global literature provides strong evidence of the success of FP interventions, to our knowledge, no systematic review has specifically generated evidence on experimentally evaluated FP interventions that had proven to improve the use of modern contraceptives in the diverse South Asian region. Our primary goal was to generate robust evidence on the effective FP programmatic interventions to influence policies and practices. The scope of this systematic review was to collate FP interventions in South Asia by synthesizing evidence solely from published experimental research (nonrandomized and randomized trials) to establish clear, causal relationships between interventions and outcomes, which is crucial for developing effective, evidence-based health policies and programs. Specifically, we aimed to estimate the effect of FP interventions on the uptake of modern methods of contraception to identify the most effective FP interventions implemented in the region. In addition, we explored the impact of interventions varied by possible intervention type (classified as demand-side, health system integrated programs, and social franchised clinics). We also assessed the impact of the urban-rural divide on intervention effectiveness to explore any existing geological disparity. Policymakers and funding organizations may use this review to support their decisions to prioritize strategies while investing in FP interventions in South Asia.

## Methods

This systematic review is registered in the International Prospective Registry of Systematic Reviews (PROSPERO) with the registration number (CRD42021262376) [26]. We strictly followed the Preferred Reporting Items for Systematic Reviews and Meta-Analyses (PRISMA) guidelines to develop the review [27].

### Search strategy

We searched databases including PubMed/MEDLINE, Cochrane, EBSCO CINAHL (Cumulative Index to Nursing and Allied Health Literature), Web of Science (WoS), and ProQuest Theses & Dissertations. The search strategy included the combination of terms and synonyms of (‘modern contraceptive use’ OR ‘modern contraceptive prevalence’ OR ‘modern method’) AND (‘women’ OR ‘reproductive age women’) AND (‘South Asia’) used as keywords in the title and abstract. (Supplementary file, Appendix A). We systematically searched peer-reviewed experimental studies conducted in South Asian countries and included those published between January 1st, 2000 and May 4, 2021. A second search was conducted from May 5, 2021, to May 4, 2023, to include more recent studies for a comprehensive and up-to-date review. The final database search was completed on August 28, 2023. This search was conducted to identify relevant studies assessing the impact of effective FP interventions among women of reproductive age (15–49 years) from countries situated in the South Asian region. The timeframe was selected with the rationale that significant progress has been made in the past two decades after the revolutionary Cairo Conference held in 1994 [26]. In response, countries increased their efforts in implementing FP interventions to meet the commitments of the Millennium Development Goals, Family Planning 2020, and Sustainable Development Goals 3 (Good Health and Well-being) and 5 (Gender Equality) by improving contraception needs and tackling reproductive health challenges [27]. Hence, this period was selected to maximize relevant literature outputs.

Apart from the studies extracted from the databases mentioned above, a manual search of reference lists from the relevant systematic reviews was also conducted to locate any further studies using the snowball technique. We also searched Google Scholar for grey literature (for experimentally evaluated program evaluations). We contacted the authors of the included studies to request any additional information or clarification on the study methodology or results when needed.

### Eligibility criteria

We considered experimental studies from South Asian countries, published from January 2000 to May 4, 2023, which compared the use of modern contraceptives among women of reproductive age (15–49 years) who received an intervention on FP versus those who received routine/standard care without any intervention. Our review encompassed studies from South Asian countries, namely Afghanistan, Bangladesh, Bhutan, India, Maldives, Nepal, Pakistan, and Sri Lanka, which were included in the review to cover the whole South Asian region. The studies included randomized control trials (RCTs), cluster-randomized trials (CRTs), quasi-experimental studies, controlled before-after (CBA) studies, and program evaluation with control groups. The study population included all women of reproductive age (15–49 years), as per the WHO definition [28] who may or may not have initiated modern contraceptive use.

The primary outcome was modern contraceptive use, i.e., women of reproductive age (15–49 years) who were currently using (or whose partner was using) a modern contraceptive method at a particular point in time (Supplementary file, Appendix B). Secondary outcomes included all contraceptive use (including traditional methods), total unmet need, method-specific use, knowledge (Supplementary file, Appendix B) of modern contraceptive methods, and other possible maternal and neonatal outcomes reported in the primary studies. Only studies written and published in English were included.

Reviews, commentaries, editorial reports, case series, articles without full text, duplicated studies, observational studies, and anonymous reports were excluded from the review process. While studies that lacked control groups or had alternative designs might have contained valuable information on FP interventions, their exclusion was deemed necessary. This decision stemmed from the challenges associated with accurately quantifying impacts from less methodologically sound designs, thereby ensuring higher reliability in the study’s finding.

### Selection of studies

We used Covidence software [29] to review the identified studies systematically. In the first stage, two reviewers (ZM, SF) independently reviewed titles and abstracts based on the eligibility criteria. A third reviewer group (HS, SR) resolved disagreements independently through Covidence software. In the second stage, two reviewers (ZM, SF) reviewed the full text of the identified articles. Disagreement at this stage was resolved via discussion between the reviewers and supported by a reason based on the eligibility criteria.

### Data extraction

Two investigators (AF, T.) independently extracted the data from each study using a data extraction template. Information related to geographical location, study design, sample size, study setting, duration of intervention, type of intervention, and adjusted effect estimates of primary and secondary outcomes was extracted. Authors of relevant articles were also contacted for any unreported data essential for the analysis, where necessary. Articles assessing multiple outcomes were restricted to only those applicable to the review’s scope. The data extraction accuracy was maintained by matching the data extracted by each investigator, and disagreements were resolved either by consensus or by involving a third additional independent investigator (HS, SR, ZM).

### Study methodological quality

Two investigators (ZM, T.) independently evaluated the studies’ methodological quality using standardized tools. For non-randomized experimental studies, we used the risk of bias in non-randomized studies – of interventions (ROBINS-I) based on seven risks of bias domains. Each domain consists of signaling questions, leading to domain-level judgment [30] Similarly, the quality of randomized studies was assessed using the Cochrane Risk-of-Bias tool for randomized trials (RoB-2). RoB-2 examined each study on five domains. Similar to ROBINS-I, each domain of RoB-2 also consists of signaling questions that lead to domain-level judgment [31].

The quality of the outcomes was assessed using the GRADE; Grading of Recommendations Assessment, Development, and Evaluation Working Group approach [32]. The tool is based on five domains: risk of bias, imprecision, inconsistency, indirectness, and publication bias. These domains are rated on four levels: very low, low, moderate, and high. Two authors rated the quality of the evidence, and disagreements were reconciled after discussion.

### Meta-analysis

We conducted the meta-analysis using Cochrane Review Manager (RevMan) software version 5.4.1 [33]. A random effect generic inverse variance weighted model was used to pool individual adjusted odds ratios (ORs) with 95% confidence intervals. This approach was chosen because most FP interventions were quasi-experimental without randomization, and collecting unadjusted estimates might have skewed the results due to baseline variations in demographic characteristics. Adjusted estimates from individual studies could provide more valid results for intervention effects. For studies where the authors did not calculate estimates, we calculated the ORs using SAS 9.4 with baseline modern contraceptive use adjustment. Furthermore, we also checked whether effect sizes from studies that included clusters were adjusted for the clustering effect by the authors. If such adjustments were not made, we applied Cochrane cluster adjustment analysis to adjust the estimates for these studies [34].

The heterogeneity between studies and subgroups was calculated using the I-squared (I^2^). Subsequently, the chi-square test was used to assess subgroup differences. The pooled effects were visualized and presented using forest plots for overall estimates and subgroup analysis estimates. We performed ‘Eggers’s test and funnel plots for the primary outcome to account for publication bias [35].

### Sensitivity analysis

A sensitivity analysis was performed to evaluate the overall effect estimate of the primary outcome from moderate-quality studies [36]. The two risk of bias domains, namely missing data and confounding, were chosen for analysis due to their potential threat to the study’s internal validity. Each study was rated at high risk if the number of participants missing from either the intervention or control group was more than 20% (80% participation criteria) or if the potential confounders (particular to each study) were not adjusted at the analysis level [37].

### Deviation from protocol

We deviated from the protocol to include data on method-specific modern contraceptive use. The reason for this addition was to fully understand which specific methods were more accepted and utilized alongside overall modern contraceptive method use. Further, limited data in studies on maternal and newborn health indicators generated no evidence for these secondary outcomes. We also did not perform the age subgroup analysis because none of the studies provided age-disaggregated estimates for modern contraceptive use.

## Results

**Fig. 1:**
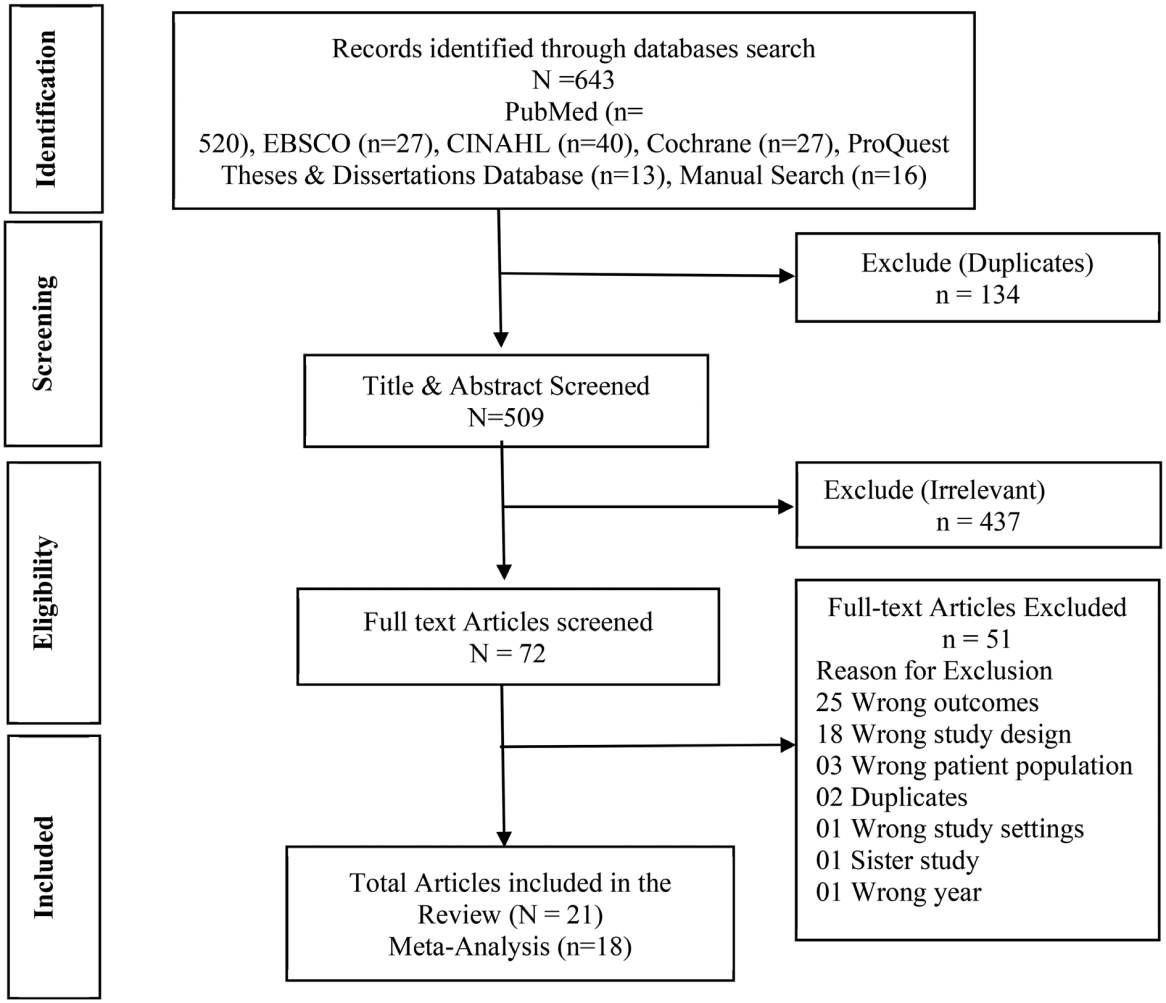
PRISMA flow chart for the study eligibility

The database search yielded a total of 643 studies. After removing 134 duplicate studies, the remaining 509 were screened for title and abstracts. Subsequently, 437 irrelevant studies were excluded from further consideration. Following a full-text review of the remaining 72 studies, 51 records that did not meet the inclusion criteria were removed. A total of 21 studies [22, 40–59] met our inclusion criteria. Out of these, three studies were sourced from grey literature, with one obtained from Google Scholar and two identified from the reference lists of relevant articles. Among the 21 included studies, 18 [40–57] were compatible for meta-analysis. However, the remaining three studies [22, 58, 59] were excluded from the meta-analysis due to disparities in outcome measurements and study designs that prevented meaningful comparison. Nonetheless, the narrative synthesis incorporated findings from all eligible studies (Fig. 1) to provide a comprehensive analysis.

### Study characteristics

The characteristics of the included studies in the review and meta-analysis were summarized (Supplementary file, Appendix C). The 21 studies combined 51,676 total participant women of reproductive age (15–49 years), and the sample size per study ranged from 500 to 5,000 participants. Apart from female participation in FP interventions, four studies encouraged male involvement. Across included studies, the mean age of women ranged from 31 to 37 years, and only two studies targeted adolescent and young married couple groups. Individual study duration varied from 9 months to 5 years, during which pre-planned intervention activities were carried out. Of the included studies, four were conducted in Bangladesh [38–41] eight in India [42–49] seven in Pakistan [50–56]and two in Nepal [57, 58]. The majority of the experimental studies (n = 18) were quasi-experimental designs [38–44, 46–55, 58] alongside two randomized controlled trials [53, 57] and one step-wedge design [58]. Among the studies from South Asian countries, 13 were predominantly community-based, four were both community and facility-based, and four were facility-based (Table 1). However, due to overlap between community and facility-based interventions, it was not feasible to undertake a meta-analysis for community based subgroup. These interventions varied widely, from facility-based counseling, as demonstrated by India’s CHARM study [45], to community-based efforts led by change agents and facilitators, exemplified by India’s PRACHAR project [42] and Bangladesh’s PLA program [39]. Pakistan adopted a multifaceted approach, combining social franchise programs, voucher schemes, and the Suraj and CMW models, targeting women of various age groups [52]. Nepal employed mass media campaigns and female community health volunteers, while Saifuddin Ahmed’s study focused on integrating family planning counseling with ongoing maternal and child health activities [41]. These initiatives collectively aimed to bolster modern contraceptive use, enhance knowledge of contraceptive methods, and diminish unmet needs among women of reproductive age, thereby contributing to the overall enhancement of reproductive health outcomes in the region.

**Table 1:** Pre-dominant classification of interventions based on settings

Health Facility Based Interventions	Community-Based Interventions	Combination
• Social franchising • Demand-side financing • Integrating FP services with existing reproductive health services • Franchised Family Planning Clinics	• Social environment building • Information sharing on reproductive health and services • Improving access to reproductive health services • Individual capacity building • Vocational Skills Training • Social mobilization • Participatory learning and action • Adolescent clubs • One-on-one counseling, discussion groups • Educational campaign • Focus group ( youth groups) • Married Adloscent Girls (MAG) clubs	• Single-purpose voucher • Multipurpose-purpose voucher (integration model) • Outreach through the Lady Health Worker Program coupled with doorstep FP services • Family planning and gender equity (FP + GE) counseling at the facility

Modern contraceptive use was reported in 15 studies [38–40, 42–45, 50–57] and 12 studies assessed overall contraceptive prevalence use inclusive of traditional methods (withdrawal, periodic abstinence, etc.). The most commonly reported secondary outcomes were knowledge of various modern methods, methods specific use, and unmet need. Limited data was reported on pregnancy, maternal, and neonatal outcomes (Table 1).

### Intervention characteristics and categories

The reported FP interventions varied widely in their components, which contributed to an increase in modern method uptake. Most interventions were delivered as packages combining different strategies to tackle barriers to modern contraceptive use. This makes classifying them into exclusive groups complex because of an overlap between their functional definitions.

However, we identified that most (n = 14) of the implemented interventions fell under the demand-generation category and were based on the behavior change theory aimed at improving knowledge about contraceptive methods, attitudes towards fertility and family size, and practices to enhance contraceptive uptake at both the individual level and community level. Interventions included under the demand-generation category included community-based health communications (n = 6), door-to-door educational (sexual and reproductive health) material distribution (n = 4), counseling by trained workers (n = 11), educational campaigns (n = 5) using mass media (n = 3) and print media (n = 2), establishing/introducing community groups (e.g., PARCHAR, Better Life Option) where women could discuss their reproductive health problems and strategize effective solutions through information sharing (n = 2), and introduction of married adolescent groups (MAD Club) where interpersonal communications between married and adolescent community could help them learn from experiences collectively (n = 1).

A second category was based predominantly on facility-based interventions (n = 4) that utilized the existing health system (either public or private) to improve accessibility, availability, and affordability through interventions targeting supply chain, quality of care, provider training to promote informed method choices and reduced costs. Included interventions involved the distribution of free vouchers for IUD insertion (n = 2) and removal along with counseling sessions (n = 1), expansion of community health worker services in the community for the provision of FP commodities (n = 1) along with other maternal care services, and the integration of FP services into ongoing maternal, neonatal, and child health (MNCH) interventions (n = 2).

A final category included three interventions that evaluated franchised FP clinics established specifically to provide services through their own network using trained community workers who counseled women to access available FP commodities at these specific clinics (e.g.,SURAJ clinics), thereby improving accessibility and affordability. This involved either the establishment of new clinic facilities with trained staff or the registration of private clinics under the franchise name to provide sexual and reproductive services.

### Modern method use and unmet need

**Fig. 2:**
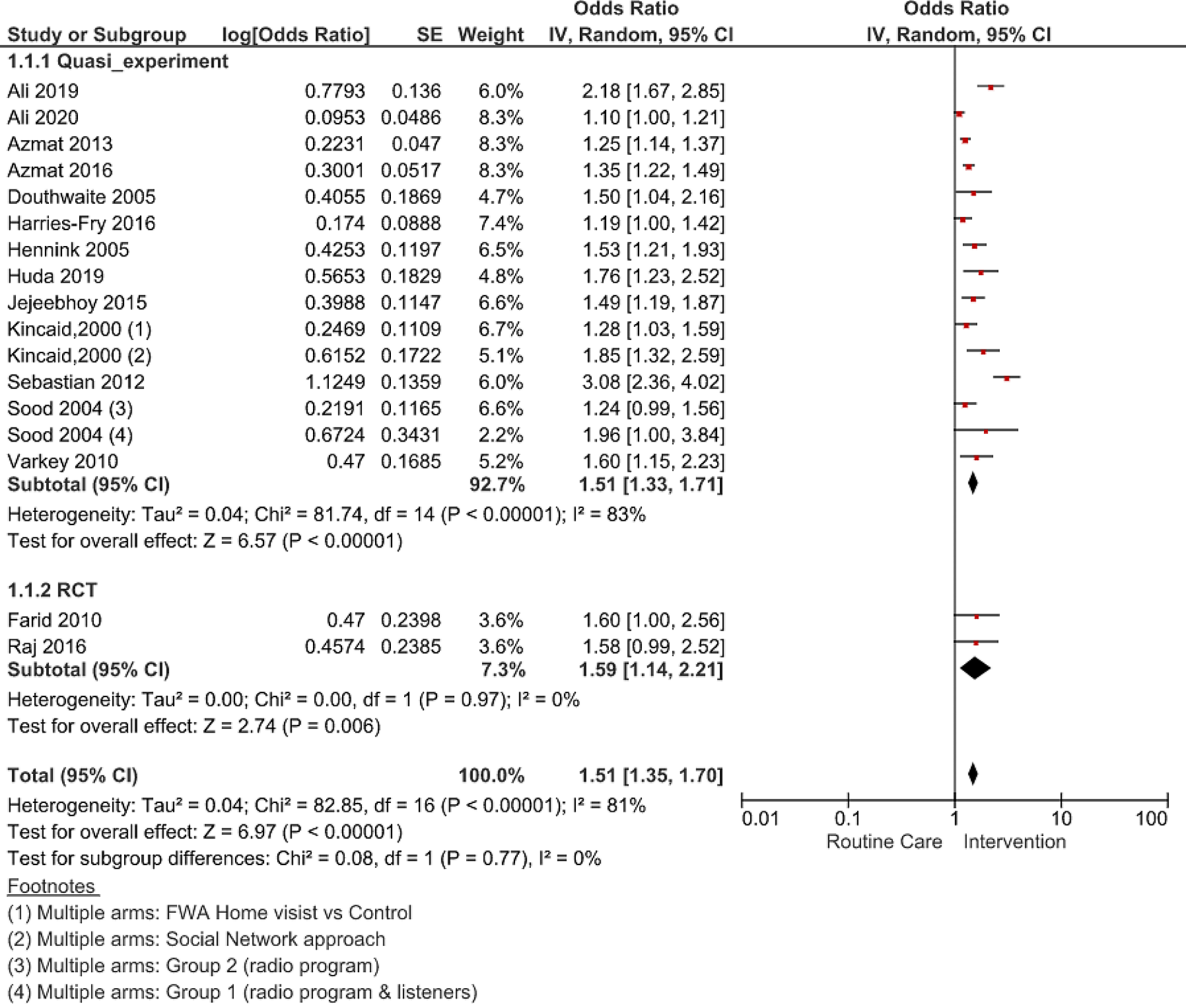
Forest plot of pooled estimate of modern contraceptive use with referenced individual studies

Fifteen studies [38–40, 42–45, 50–57] that reported the impact of interventions on the uptake of modern contraceptives among women of reproductive age (15–49 years) were included in the meta-analysis (thirteen quasi-experimental studies and two cluster RCTs). The meta-analysis results revealed significantly higher odds of modern contraceptive use (pooled OR 1.51; 95% CI 1.35–1.70, p-value < 0.00001, I^2^ = 81%, GRADE: very low) the intervention areas when compared to routine care in comparison groups (Fig. 2).

The most commonly reported and adjusted covariates in individual studies were age, education, number of children, socioeconomic status, and baseline modern contraceptive use. Given the significant heterogeneity in effect sizes between the studies, we identified that quasi-experimental studies were the main source of variability (within group I^2^ = 83%), which was further explored in the subgroup analysis. Due to high heterogeneity, caution must be exercised when interpreting the adjusted pooled estimates for interventions’ impact.

The overall contraceptive use (inclusive of all methods) reported in 12 studies and pooled estimates showed a positive impact of the interventions (OR 1.73; 95% CI: 1.51–1.98, p-value < 0.00001, I^2^ = 83%). Though only three studies presented data on unmet need for contraception, the pooled effect was significant, with a 14% total reduction in unmet need attributed to FP interventions (OR 0.86; 95% CI: 0.78–0.94, p-value < 0.002, I^2^ = 50%).

### Subgroup analysis: effect of intervention type on modern method use

**Fig. 3:**
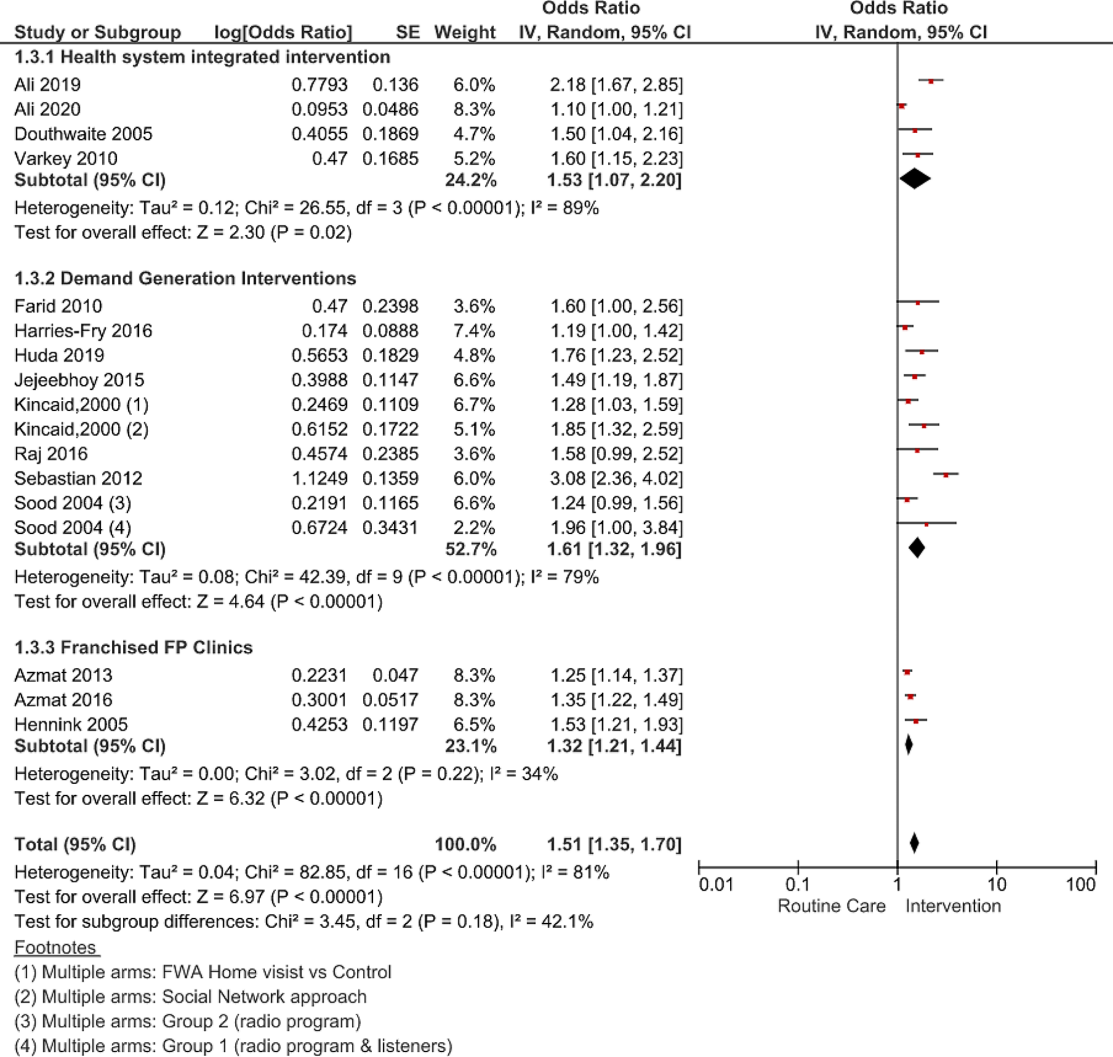
Forest plot pooled estimates for modern contraceptive use by intervention type and referenced individual studies

A subgroup analysis stratified by intervention type (Fig. 3) observed that pooled estimate for demand-generation interventions (n = 8) was significantly the highest (OR 1.61; 95% CI: 1.32–1.96, p-value < 0.00001, I^2^ = 79%), followed by existing health system integrated (n = 4) interventions (OR 1.53; 95% CI 1.07–2.20, p-value < 0.02, I^2^ = 89%), and franchised FP clinic interventions (OR 1.32; 95% CI 1.21–1.44, p-value < 0.00001, I^2^ = 34%). Each of these intervention categories has been demonstrated to be effective in South Asia, albeit with varying levels of impact. Notably, demand-side interventions, which typically incorporated educational and behavioral approaches, had shown a high potential for increasing modern contraceptive use compared to other intervention categories.

While exploring the geographical locations within South Asia where these interventions were implemented, all three franchised FP clinic intervention studies were conducted in Pakistan. The majority of demand-generation interventions were reported from India and Bangladesh, while the health system integrated model was tested in most of the countries; hence, when interpreting, it is important to consider the context of implementation, especially geographical region, the standard of routine care, offered in each country against which the intervention effect is evaluated.

### Effect of urban-rural divide on modern method use

**Fig. 4:**
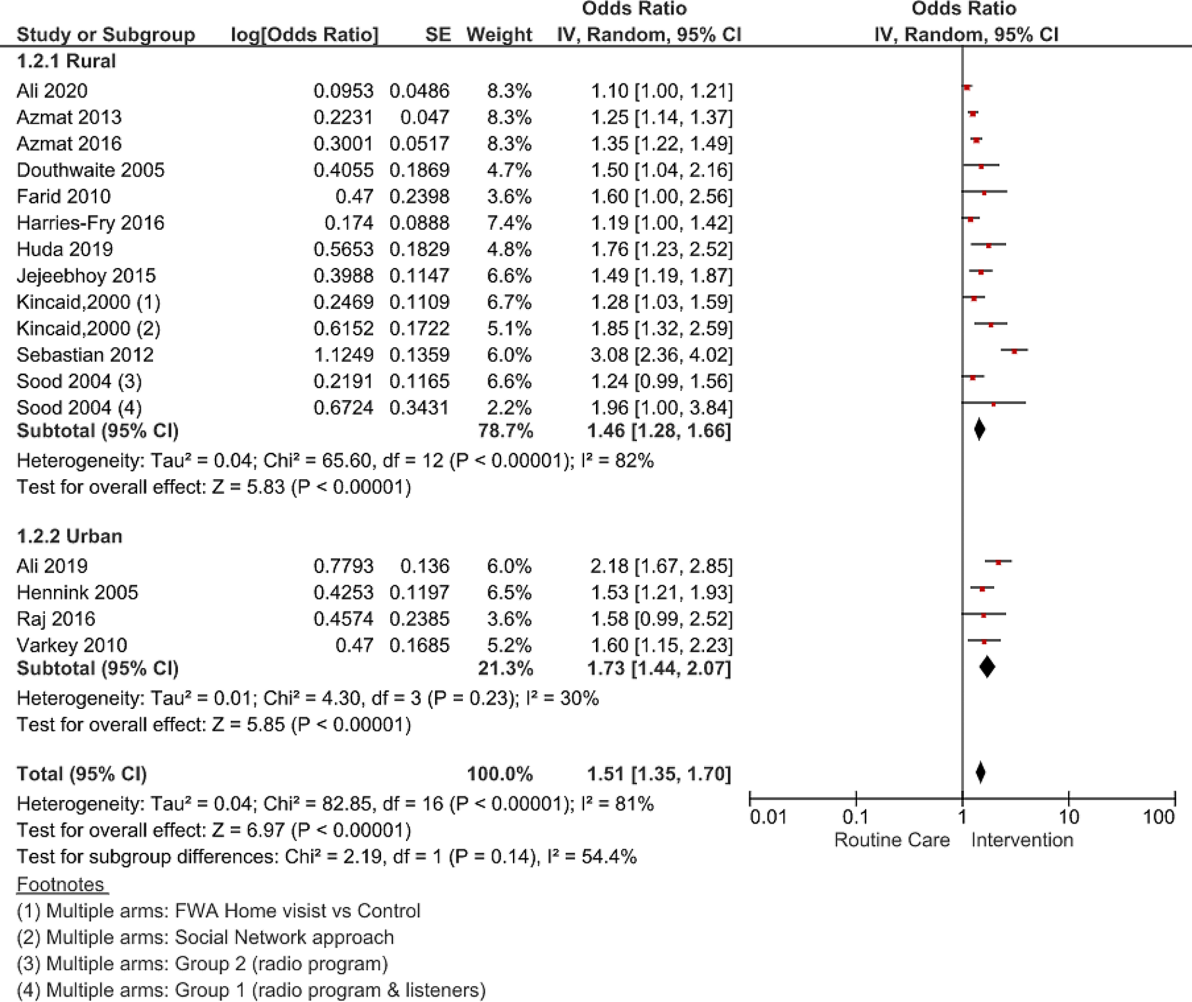
Forest plot pooled estimates for modern contraceptive use by urban-rural divide and referenced individual studies

Eleven studies were conducted in rural settings, while four were carried out in urban areas (Fig. 4). The pooled results revealed substantial variation in the modern contraceptive use estimates by urban-rural stratification. Modern contraceptive use was significantly higher in urban settlements (OR 1.73; 95% CI 1.44–2.07, p-value < 0.00001, I^2^ = 30%) as compared to rural settings (OR 1.46; 95% CI 1.28–1.66, p-value < 0.00001, I^2^ = 82%). A large part of the heterogeneity in the studies conducted in rural areas when compared to urban areas was explained by differences in the intervention types, area demographics, and study quality.

### Country level subgroup analysis on effect of FP interventions on modern method use

**Fig. 5:**
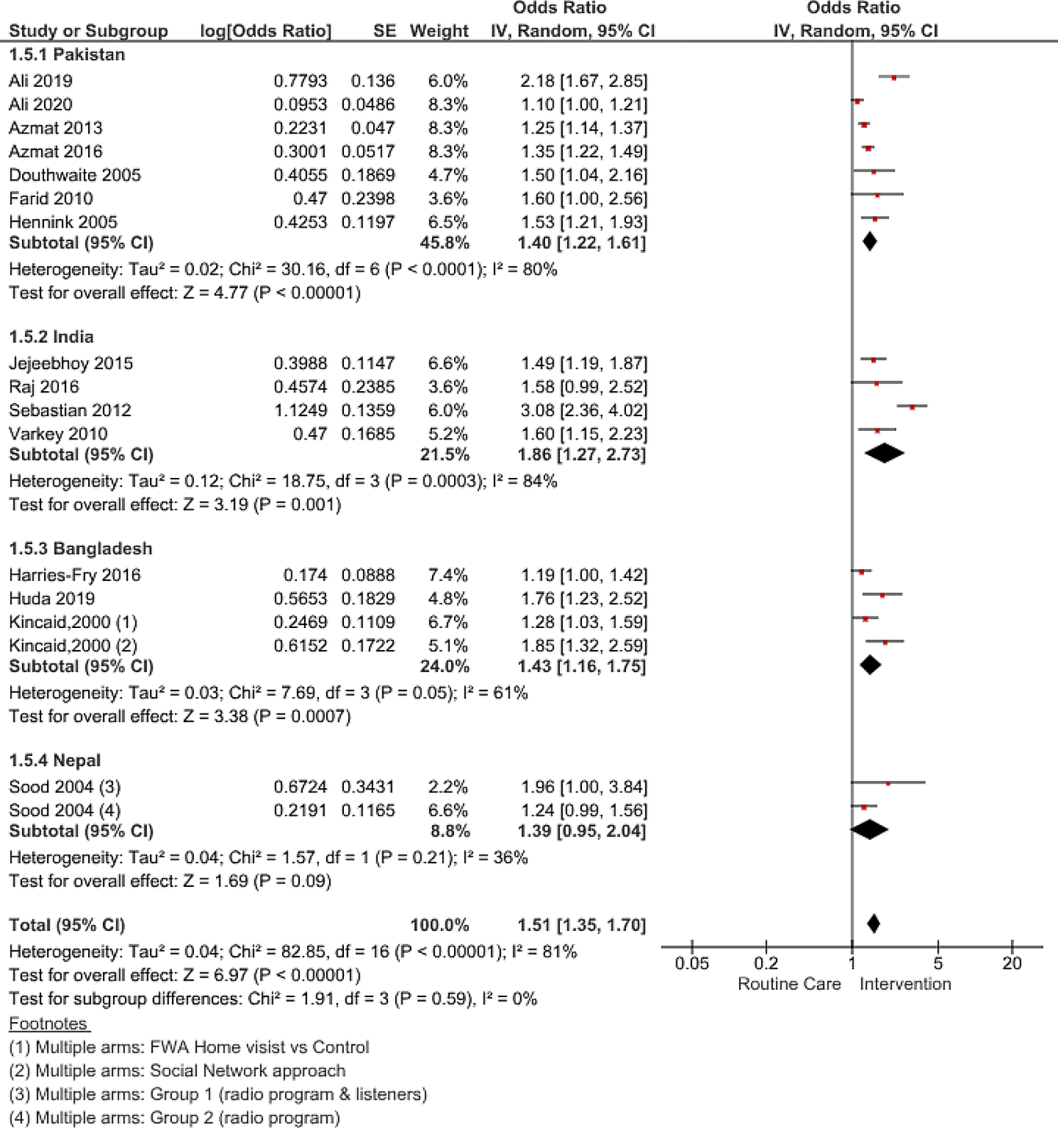
Forest plot pooled country disaggregated estimates for modern contraceptive use

A country stratified analysis of the FP intervention for improving the use of modern contraceptive showed significant estimates for India (OR 1.86; 95% CI 1.27–2.73, p-value = 0.0011, I^2^ = 84%) Bangladesh (OR 1.43; 95% CI 1.16–2.75, p-value = 0.0007, I^2^ = 61%) and Pakistan (OR 1.40; 95% CI 1.22–1.61, p-value < 0.00001, I^2^ = 80%). However, results for Nepal (OR 1.39; 95% CI 1.44–2.07, p-value = 0.09, I^2^ = 36%) were not significant (Fig. 5).

### Modern method knowledge

The meta-analysis pooled OR for knowledge about available contraceptive methods is presented in Table 2. The increase was significant for a few modern methods in FP intervention groups compared to the routine care group. Specifically, there was a significant increase in knowledge of condoms (OR 2.03; 95% CI 1.19–3.47, p-value = 0.009), pills (OR 1.45; 95% CI 1.01–2.08, p-value = 0.04), injectable (OR 1.55; 95% CI 1.03–2.33), p-value = 0.03), implants (OR 2.86; 95% CI 1.16–7.06), p-value = 0.02), and female sterilization methods (OR 1.59; 95% CI 1.03–2.45, p-value = 0.04). However, the OR for male sterilization and intrauterine devices (IUDs) were insignificant. This lack of significance can be attributed to already prevalent knowledge of male sterilization and IUDs in both the intervention and control groups; hence, no pronounced increase was observed.

**Table 2:** Results of meta-analysis on the impact of FP interventions on secondary outcomes

Outcome	Number of studies	Odds Ratio (95% CI)	Heterogeneity	
			Chi^2^ p-value	I^2^ (%)
All Contraceptive method use	12	1.73 (1.51–1.98)	0.00001	83
Total unmet need	3	0.86 (0.78–0.94)	0.14	50
**Current use of**				
Pills*	11	1.38 (1.06–1.79)	0.00001	75
Condom*	11	1.32 (1.0, 1.77)	0.00001	93
Injectable	8	0.92 (0.67–1.27)	0.00001	88
Male sterilization	4	1.22 (0.73–2.05)	0.16	37
Female Sterilization	9	0.91 (0.71–1.15)	0.001	82
Intra-Uterine Device*	11	1.62 (1.05–2.50)	0.001	91
Implants	4	1.76 (0.86–3.61)	0.11	44
**Knowledge**				
Condom*	7	2.03 (1.19–3.47)	0.001	98
Pills	8	1.45 (1.01–2.08)	0.001	95
Injectable	6	1.55 (1.03–2.33)	0.001	97
Male sterilization	6	1.63 (0.81–3.29)	0.001	99
Female Sterilization	6	1.59 (1.03, 2.45)	0.001	97
Intra-Uterine Device	6	1.53 (0.79–2.95)	0.001	99
Implants	3	2.86 (1.16–7.06)	0.001	97
ECP use	3	1.31 (0.65–2.67)	0.001	91

^*^are the outcome variables with P-value of random-effect model is < 0.05

### Maternal and neonatal outcomes

We found that only two [43, 56] studies reported improvements in maternal and neonatal health outcomes. One RCT [56] reported a 10% reduction in neonatal mortality and a 7% increase in antenatal care visits in the intervention group compared to the control group. However, no comprehensive results could be deduced due to the limited data available for the studies.

### Method specific use

Though method-specific use was not mentioned in the protocol, we deviated to include additional outcomes on method-specific use to gain a more holistic picture. Exploring method-specific knowledge and identifying which modern methods were opted for by participant women in response to FP interventions is important evidence that should be generated to inform future policy and intervention design.

We pooled the estimates for commonly used modern contraceptive methods presented in Table 1. Among them, IUDs had the highest significant increase (OR 1.62; 95% CI 1.05–2.50, p-value = 0.03) followed by pills (OR 1.3; 95% CI 1.06–1.79 p-value = 0.02) and condoms (OR 1.32; 95% CI:1.0-1.77, p-value < 0.05). Pooled estimates for other methods, including implants, male sterilization, female sterilization, and injectables, were statistically not significant.

### Risk of bias and sensitivity analysis

The risk of bias assessment was undertaken for all the 21 studies reported in supplementary file Appendix D. The eighteen studies included in our meta-analysis, sixteen quasi-experimental were evaluated for risk of bias using the ROBINS-I tool, which is suitable for non-randomized experimental studies. Of these studies, ten were assessed to have a moderate risk of bias, seven were deemed to have a high risk of bias, and none were considered to have a low risk of bias. The two RCTs were assessed using the Cochrane RoB-2 tool and were found to be at high risk of bias due to limited information on the authors’ randomization process. The remaining three studies, which were part of the narrative but not included in the meta-analysis due to design and outcome assessment differences, were found to be at high risk on ROBINS-I tool (Supplementary file, Appendix D).

Because most FP-related interventions were quasi-experimental, confounding variables were identified as a potential area of bias across all studies, particularly in the quasi-experimental studies, due to unknown and residual confounding at the design level. The highest risk of bias percentages was associated with confounding and incomplete data domains, highlighting the need for sensitivity analysis (Supplementary file, Appendix E) to detect high-risk studies that could influence the primary outcome. After omitting the high-risk studies based on confounding [39, 44, 51, 52] and incomplete data domain [56] the sensitivity analysis results remained significant (OR 1.50; 95% CI 1.28, 1.77; p-value < 0.00001).

**Fig. 6:**
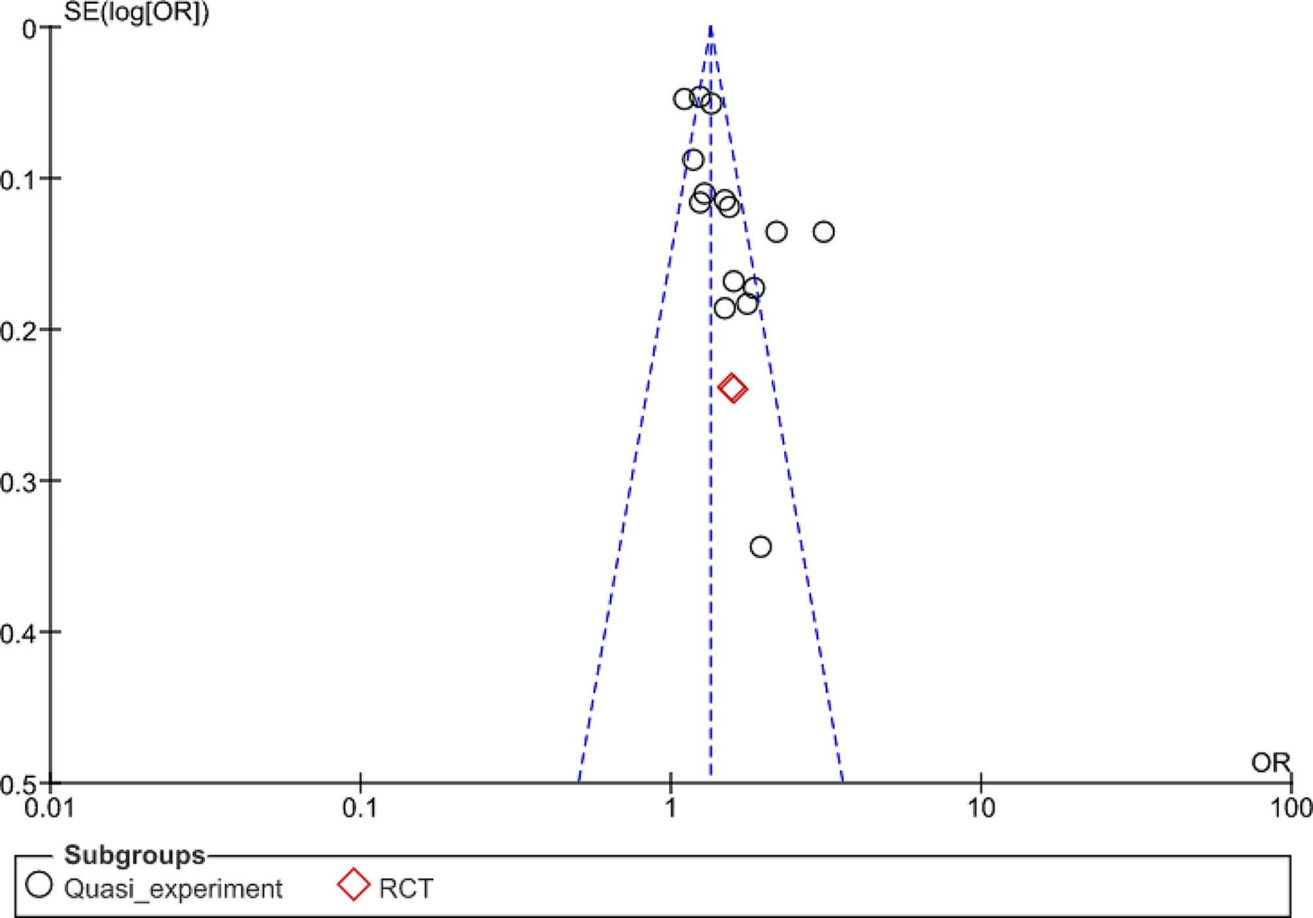
Funnel plot for assessing publication bias on modern contraceptive sue

To assess publication bias and the effect of small studies, we generated a funnel plot for modern contraceptive use outcome (Fig. 6). The funnel plot asymmetry indicated that publication bias could affect the reliability of our results. It emphasized that only those interventions that had improved modern contraceptive use were reported in the literature. Furthermore, there may be other interventions that have not been studied through experimental research were not included. Moreover, Egger’s test (t = 2.49, p = 0.027) indicated publication bias in reporting results. Therefore, based on these results, it can be interpreted that there is evidence of publication bias in the meta-analysis being investigated. Our GRADE analysis for modern contraceptive use showed very low quality (Supplementary file Appendix F), primarily due to the quasi-experimental design of most studies, which lacked randomization. Nonetheless, the evidence was still considerable.

## Discussion

This systematic review and meta-analysis estimated the impact of different FP interventions on the uptake of modern methods of contraception in South Asia among women of reproductive age (15–49 years). The meta-analysis revealed that FP interventions significantly impact the uptake of modern contraceptive methods in the region. Specifically, women in the intervention areas were more likely (pooled OR 1.51) to use modern contraceptives than women in the control groups who received only routine care in their respective regions.

Furthermore, the knowledge of modern methods was improved in intervention groups where participant women could name relatively more modern methods and were aware of their utility and respective side effects. Alongside, there was a significant 14% (pooled OR 0.86) reduction in unmet need among the participant women due to effective FP interventions. Similar results were reported in a meta-analysis that specifically targeted only postpartum women, with a significant increase in modern contraceptive uptake of 41.2% (95% CI: 15.7–69.1%) observed attributed to FP interventions. Most of these programs included in this review were implemented in East and West Africa and South Asia [59]. These findings suggest that FP interventions are effective not only when targeted only at postpartum women but also for all women of reproductive age (15–49 years) for improving modern contraceptive use.

One of the practical considerations from the review is related to the intervention type. Outcomes such as knowledge, attitudes, FP use, and reduction in unmet need were improved due to FP interventions. However, the review findings suggest that demand-side interventions have a significantly higher impact (pooled OR 1.61, p-value < 0.00001) on modern contraceptive uptake. Our evidence is also supported by a meta-analysis of 14 demand-generation interventions implemented in LMICs, which showed a positive association with modern contraceptive use (pooled OR 1.57, p-value < 0.01). Further, their subgroup analysis of intervention types also suggested that demand-side interventions are diverse in nature and operations with the capacity to influence ‘society’s contraceptive behaviors and attitudes and could serve as evidence to bring constructive change [22]. For example, one study that implemented mass media interventions revealed a positive impact on contraceptive usage and unmet need; interpersonal communication interventions also showed similar benefits [60].

Besides demand-generation, our review further reported health system strengthening FP services that resulted in significant improvements in modern method use. These results aligned with WHO guidelines, which signify that the MCH/FP package is considered a necessary condition for the program’s success in South Asia. This translates into integrating FP services within the existing MNCH service frameworks to capture missed opportunities. Additionally, another systematic review examined that voucher interventions integrated into the existing healthcare system can improve knowledge, service utilization, and quality [56]. However, the pooled estimates for demand-generation interventions were higher than the health system interventions reported in our study. This affirms the assumption that if FP services and supplies are available and accessible, generating demand for contraceptive use is imperative to achieve high modern contraceptive prevalence rates.

Furthermore, some individual studies assessed that residing in rural areas is negatively associated with modern contraceptive use [13] additionally, urban residents are more likely to use modern contraceptives voluntarily [61]. In countries with significant poverty, low literacy, and higher rural populations, studies have shown that reforming health systems improves FP behaviors, boosts FP practices, and reduces unmet need [62]. Though the findings of our meta-analysis showed that FP interventions increased the uptake of modern methods in rural areas, the impact is significantly more profound in urban localities. These variations can relate to sociodemographic and economic factors or the disproportionate healthcare services offered in rural and urban environments [13].

It is also worth noting that even if the outcomes were favorable, they were not always constant across multiple locations or target groups. In Nicaragua, for example, the voucher program had differing impacts on school-aged adolescents who accessed the program in an urban setting as compared to those who accessed the program in a rural community-based setting [63]. Thus, a strategic and integrated approach can help target barriers to contraceptive accessibility, affordability, and acceptability.

Apart from improving modern method use and reducing unmet need, our results showed that the interventions increased knowledge of specific contraceptive methods, including the advantages and disadvantages of each method. Increased knowledge also translated into a better understanding of and generated demand for long-term contraceptives over shorter-term methods, as evidenced by an increase in implant and condom knowledge along with their use in our review.

The increase in modern method use is attributed to an increase in knowledge about particular methods. This may promote informed choices by allowing women to recognize that more methods are available to meet their needs. Another important finding is the increased use of IUDs in the intervention group where women were already knowledgeable. These results explain that FP interventions can reduce barriers to an ‘individuals’ fear and address community stigmatization by improving their acceptability through educational programs.

### Limitations

One limitation of our review is that it only focused on experimentally evaluated studies with a control group in order to obtain pooled estimates with reduced variability. This strategy then limited our ability to collect data on many interventions implemented in the region that used other designs, such as pre-post designs without control or observational studies. The studies included in this meta-analysis were predominantly non-randomized quasi-experimental trials. The heterogeneity was very high, and the GRADE analysis presented very low certainty. Some aspects of heterogeneity were explained in the subgroup analysis, but there were some overlaps in classification due to the complexity of most family planning interventions, making it difficult to assess individual intervention components. To ensure evidence-based results, we selected studies with control groups. However, routine care may vary across countries and regions, which could affect the assessment of interventions in individual studies and, hence, the pooled estimates. This limitation raises concerns about the reliability and generalizability of the findings related to modern contraceptive use.

### Implications

Our review findings signify that FP programs should embed demand-generation interventions as an integral part of any FP service delivery model, especially in South Asian regions. Demand-generation FP interventions that focus on addressing individual and community-level barriers and socio-cultural norms have a high potential to increase the use of modern contraceptives and reduce the unmet need to meet set reproductive goals. However, other FP interventions that could be incorporated into the existing healthcare system or opening new franchising are also effective when accessibility is the main focus.

The higher level of acceptance towards FP uptake in urban compared to the rural environment in response to FP interventions in South Asia highlighted existing geographical disparities and inequities. Nonetheless, these findings also emphasized the importance of an enabling environment that can provide the opportunity for interventions to perform and produce better outcomes. There is a need to invest in improving female literacy, empowerment, and other infrastructure, which can lead to improved access and uptake of FP services. These investments should be targeted and tailored, especially for rural settings, to promote a conducive environment for intervention implementation, improving reproductive maternal and child health outcomes and reducing existing urban-rural disparities.

## Conclusion

To our knowledge, this is the first systematic review of experimental studies that estimated the impact of different FP programs on the uptake of modern methods of contraception in South Asia and identified effective FP strategies. The meta-analysis reveals that FP interventions had a significant impact on improving modern contraceptive use. It demonstrated the effectiveness of different approaches, extending beyond the demand generation. Strategies integrating FP into health system and franchised FP clinic models were particularly successful in the South Asian context. This stratification offers crucial insights into the effectiveness of strategies specific to different contexts. Further, the uptake of the modern methods of contraceptives was significantly higher in urban areas as compared to rural areas, emphasizing existing disparities. It emphasizes the necessity to tailor FP programs for rural contexts to achieve more significant impact. This underscores that the success of interventions is influenced by the supportive environment in which they are implemented, addressing a critical gap in understanding the pivotal role of an enabling environment in achieving the impacts of FP interventions. Hence, policymakers and researchers should consider these aspects when designing interventions and programs, particularly considering the contextual differences. Understanding what works for whom and in which setting is crucial in planning and implementing cost-effective and successful programs. The review also uncovered a notable research gap; the limited evaluation of maternal and neonatal outcomes in FP interventions, indicating a need for future studies to explore the broader implications of FP on maternal and child health along with modern contraceptive method use. Further innovation and delivery models need to be tested in South Asia to expand our evidence base for interventions targeting FP-related outcomes.

### Electronic supplementary material

Below is the link to the electronic supplementary material.

**Supplementary File 1:** PRISMA abstract checklist

**Supplementary File 2:** PRISMA checklist

**Supplementary File 3:** Details of search strategy, individual study characteristics tables, risk of bias assessment and sensitivity analysis for the systematic review and meta-analysis

## Data Availability

The datasets analyzed as part of this review are available from the corresponding author on request.

## Abbreviations

FP: Family Planning
OR: Odds ratio
CI: Confidence interval
GRADE: Grading of Recommendations Assessment, Development, and Evaluation
PRISMA: Preferred Reporting Items for Systematic Reviews and Meta-Analyses
I^2^: Heterogeneity
