# Supplementary material for: Effective strategies for increasing the uptake of modern methods of family planning in South Asia: a systematic review and meta-analysis
Source: BMC Womens Health. 2024 Jan 3;24:13. doi: 10.1186/s12905-023-02859-2 (PMC10765777; doi:10.1186/s12905-023-02859-2)
Supplement: Supplementary file 3 — Details of search strategy, individual study characteristics tables, risk of bias assessment and sensitivity analysis for the systematic review and meta-analysis [file 12905_2023_2859_MOESM3_ESM.docx]

|  |
| --- |
| Supplementary Files |
| ***Effective Strategies for Increasing the Uptake of Modern Methods of Family Planning in South Asia: A Systematic Review and Meta-analysis*** |

**Table of Contents**

[**APPENDIX A**: Search Strategy 2](#_Toc106983019)

[**APPENDIX B**: Panel Definitions 5](#_Toc106983020)

[**APPENDIX C**: Characteristics of the Included Studies 6](#_Toc106983021)

[**APPENDIX D**: Risk of Bias 12](#_Toc106983025)

[Figure A: Risk of Bias assessment for Quasi Experimental studies 13](#_Toc106983026)

[Figure B: Risk of Bias assessment for Randomized Control Trials 14](#_Toc106983027)

[**Appendix E**: Sensitivity Analysis 15](#_Toc106983028)

[**Appendix F:** Grade 16](#_Toc106983029)

# **APPENDIX A: Search Strategy**

**PubMed**

("Family Planning Services"[Mesh] OR "Family Planning Policy"[Mesh] OR "Sex Education"[Mesh] OR "Natural Family Planning Methods"[Mesh] OR Family planning OR Family size OR birth prevention OR conception prevention OR

"Family Planning Policy" OR "Family Planning Services" OR "Family planning intervention*" OR "Family planning strateg*" OR "Family Planning behavior*")

AND ("Contraception"[Mesh] OR "Contraception, Barrier"[Mesh] OR "Contraception Behavior"[Mesh] OR "Hormonal Contraception"[Mesh] OR "Long-Acting Reversible Contraception"[Mesh] OR "Contraception, Immunologic"[Mesh] OR "Contraception, Postcoital"[Mesh] OR Oral contraceptives OR Combined oral contraceptives OR Sequential oral contraceptive OR "Ethinyl Estradiol-Norgestrel Combination" OR Oral hormonal Contraceptives OR "Natural Family Planning Methods" OR Contraceptive Devices OR Female Intrauterine Devices OR "Intrauterine Device Migration" OR "Intrauterine Device Expulsion" OR "Medicated Intrauterine Devices" OR "Copper Intrauterine Devices" OR Condoms OR Female Sterilization OR reproductive contraceptive barrier OR Female Contraceptive Devices OR "Vaginal Creams" OR foam OR Jellies OR "Spermatocidal Agents" OR "Ovulation Detection") AND

(Transport OR family planning clinics OR visits OR community health workers OR counseling OR contraceptive methods OR surgical sterilization services OR prescription OR family planning training OR free OR subsidized OR MNCH OR incentive OR voucher* OR cash transfer* OR Advocacy OR community OR community partnership OR community leaders OR social marketing OR modern contraceptives OR text message* OR voice message* OR mobile health OR community organization* OR advocacy material* OR Mass Media OR IEC materials OR Standard of care) AND (Modern Contraceptive Prevalence Rate OR mCPR OR Unwanted births OR unintended pregnancy OR Induced abortion OR unsafe abortion OR Maternal mortality OR Adolescent pregnancy OR Inter pregnancy Intervals OR Infant mortality OR Stillbirths OR Fertility rate OR demand met OR demand satisfied OR KAP OR beliefs) AND (South Asia* OR India OR Bangladesh OR Bhutan Maldives OR Nepal OR Pakistan OR Sri Lanka OR Afghanistan)

**EBSCO CINAHL**

("Family Planning Services"[Mesh] OR "Family Planning Policy"[Mesh] OR "Sex Education"[Mesh] OR "Natural Family Planning Methods"[Mesh] OR Family planning OR Family size OR birth prevention OR conception prevention OR "Family Planning Policy" OR "Family Planning Services" OR "Family planning intervention*" OR "Family planning strateg*" OR "Family Planning behavior*") AND ("Contraception"[Mesh] OR "Contraception, Barrier"[Mesh] OR "Contraception Behavior"[Mesh] OR "Hormonal Contraception"[Mesh] OR "Long-Acting Reversible Contraception"[Mesh] OR "Contraception, Immunologic"[Mesh] OR "Contraception, Postcoital"[Mesh] OR Oral contraceptives OR Combined oral contraceptives OR Sequential oral contraceptive OR "Ethinyl Estradiol-Norgestrel Combination" OR Oral hormonal Contraceptives OR "Natural Family Planning Methods" OR Contraceptive Devices OR Female Intrauterine Devices OR "Intrauterine Device Migration" OR "Intrauterine Device Expulsion" OR "Medicated Intrauterine Devices" OR "Copper Intrauterine Devices" OR Condoms OR Female Sterilization OR reproductive contraceptive barrier OR Female Contraceptive Devices OR "Vaginal Creams" OR foam OR Jellies OR "Spermatocidal Agents" OR "Ovulation Detection") AND (Transport OR family planning clinics OR visits OR community health workers OR counseling OR contraceptive methods OR surgical sterilization services OR prescription OR family planning training OR free OR subsidized OR MNCH OR incentive OR voucher* OR cash transfer* OR Advocacy OR community OR community partnership OR community leaders OR social marketing OR modern contraceptives OR text message* OR voice message* OR mobile health OR community organization* OR advocacy material* OR Mass Media OR IEC materials OR Standard of care) AND (Modern Contraceptive Prevalence Rate OR mCPR OR Unwanted births OR unintended pregnancy OR Induced abortion OR unsafe abortion OR Maternal mortality OR Adolescent pregnancy OR Inter pregnancy Intervals OR Infant mortality OR Stillbirths OR Fertility rate OR demand met OR demand satisfied OR KAP OR beliefs) AND (South Asia* OR India OR Bangladesh OR Bhutan Maldives OR Nepal OR Pakistan OR Sri Lanka OR Afghanistan

**Cochrane Database**

("Family Planning Services"[Mesh] OR "Family Planning Policy"[Mesh] OR "Sex Education"[Mesh] OR "Natural Family Planning Methods"[Mesh] OR Family planning OR Family size OR birth prevention OR conception prevention OR "Family Planning Policy" OR "Family Planning Services" OR "Family planning intervention*" OR "Family planning strateg*" OR "Family Planning behavior*") AND ("Contraception"[Mesh] OR "Contraception, Barrier"[Mesh] OR "Contraception Behavior"[Mesh] OR "Hormonal Contraception"[Mesh] OR "Long-Acting Reversible Contraception"[Mesh] OR "Contraception, Immunologic"[Mesh] OR "Contraception, Postcoital"[Mesh] OR Oral contraceptives OR Combined oral contraceptives OR Sequential oral contraceptive OR "Ethinyl Estradiol-Norgestrel Combination" OR Oral hormonal Contraceptives OR "Natural Family Planning Methods" OR Contraceptive Devices OR Female Intrauterine Devices OR "Intrauterine Device Migration" OR "Intrauterine Device Expulsion" OR "Medicated Intrauterine Devices" OR "Copper Intrauterine Devices" OR Condoms OR Female Sterilization OR reproductive contraceptive barrier OR Female Contraceptive Devices OR "Vaginal Creams" OR foam OR Jellies OR "Spermatocidal Agents" OR "Ovulation Detection") AND (Transport OR family planning clinics OR visits OR community health workers OR counseling OR contraceptive methods OR surgical sterilization services OR prescription OR family planning training OR free OR subsidized OR MNCH OR incentive OR voucher* OR cash transfer* OR Advocacy OR community OR community partnership OR community leaders OR social marketing OR modern contraceptives OR text message* OR voice message* OR mobile health OR community organization* OR advocacy material* OR Mass Media OR IEC materials OR Standard of care) AND (Modern Contraceptive Prevalence Rate OR mCPR OR Unwanted births OR unintended pregnancy OR Induced abortion OR unsafe abortion OR Maternal mortality OR Adolescent pregnancy OR Inter pregnancy Intervals OR Infant mortality OR Stillbirths OR Fertility rate OR demand met OR demand satisfied OR KAP OR beliefs) AND (South Asia* OR India OR Bangladesh OR Bhutan Maldives OR Nepal OR Pakistan OR Sri Lanka OR Afghanistan) in Title Abstract Keyword

**ProQuest Theses & Dissertations Database**

ab((Family planning OR Family size OR birth prevention OR conception prevention OR Family Planning Policy OR Family Planning Services OR Family planning intervention OR Family planning strategies OR Family Planning behaviors) ) AND ab((Oral contraceptives OR Combined oral contraceptives OR Sequential oral contraceptive OR Ethinyl Estradiol-Norgestrel Combination OR Oral hormonal Contraceptives OR Natural Family Planning Methods OR Contraceptive Devices OR Female Intrauterine Devices OR Intrauterine Device Migration OR Intrauterine Device Expulsion OR Medicated Intrauterine Devices OR Copper Intrauterine Devices OR Condoms OR Female Sterilization OR reproductive contraceptive barrier OR Female Contraceptive Devices OR Vaginal Creams OR foam OR Jellies OR Spermatocidal Agents OR Ovulation Detection)) AND ab((Transport OR family planning clinics OR visits OR community health workers OR counseling OR contraceptive methods OR surgical sterilization services OR prescription OR family planning training OR free OR subsidized OR MNCH OR incentive OR vouchers OR cash transfers OR Advocacy OR community OR community partnership OR community leaders OR social marketing OR modern contraceptives OR text message OR voice message OR mobile health OR community organizations OR advocacy material OR Mass Media OR IEC materials OR Standard of care) ) AND ab((Modern Contraceptive Prevalence Rate OR mCPR OR Unwanted births OR unintended pregnancy OR Induced abortion OR unsafe abortion OR Maternal mortality OR Adolescent pregnancy OR Inter pregnancy Intervals OR Infant mortality OR Stillbirths OR Fertility rate OR demand met OR demand satisfied OR KAP OR beliefs)) AND ab((South Asia OR India OR Bangladesh OR Bhutan Maldives OR Nepal OR Pakistan OR Sri Lanka OR Afghanistan))

# **APPENDIX B: Panel Definitions**

| Modern Contraceptive Use | The percentage of women of reproductive age who were using (or whose partner was using) a modern contraceptive method at a particular point in time. |
| --- | --- |
| All Contraceptive use | The percentage of women of reproductive age who use (or whose partners use) any contraceptive method at a given point in time. |
| Unmet Need | Married women who are not using a contraceptive method, are fecund, and do not wish to have any more children. |
| Modern Contraceptive Methods | Only long and short acting modern methods of contraceptivs (pill, intrauterine devices, implant, condoms, diaphragm/foam/jelly, female sterilization, and/or male sterilization). |
| Modern Contraceptive Method Knowledge | Aware of 1) name of any one modern contraceptive method either on spontaneous response or on probing, 2) usage procedure, 3) side effects |
|  |  |

# **APPENDIX C: Characteristics of the Included Studies**

| **Author,**  **Publication year,**  **Country** | **Design, Intervention**  **Delivered through,**  **Duration** | **Sample size** | **Participants** | **Outcomes reported** | **Confounders** | **Risk of Bias** |
| --- | --- | --- | --- | --- | --- | --- |
| Ali, 2019,  Pakistan | Quasi-experimental  Single purpose  voucher Scheme  LHV, field health educators, and doctors  3 years | Intervention: 1318  Control: 1296 | Women (18 – 49) Years | Modern contraceptive prevalence rate  Contraceptive prevalence rate  Current use of method uses.  pill, injectable, condom, Female sterilization, IUD, Implant.  Knowledge of contraceptive methods.  Pill, Condom, female sterilization, IUD, Injectable, Male sterilization, Implants | Respondent’s age, husband’s age, respondent’s education, husband’s education, household size, baseline, and end-line survey points | Moderate |
| Ali, 2020,  Pakistan | Quasi-experimental  Multi-purpose  voucher scheme,  3 years | Intervention: 1311  Control: 1316 | Women (18 – 49) Years | Modern contraceptive prevalence rate  Contraceptive prevalence rate  Current use of method uses  Pill, injectable, condom, Female sterilization, IUD. | Respondents’ age, husbands’ age, respondents’ education, husbands’ education, household size, and socioeconomic status | High |
| Azmat, 2013,  Pakistan | Quasi-experimental  Social franchise program and vouchers for long-term contraceptive method (IUCD)  Private service providers, field worker mobilizers,  18 Month | Intervention: 2483  Control: 1984 | Women (15 – 49) Years | Modern contraceptive prevalence rate  Contraceptive prevalence rate  Current use of method uses  Pill, injectable, condom, Female sterilization, Male Sterilization, IUD.  Knowledge of contraceptives methods  Pill, Condom, female sterilization, IUD, Injectable, Male sterilization, Female sterilization, Unmet Need. | Baseline survey points, clustering | High |
| Azmat,  2016,  Pakistan | Quasi-experimental  a) Suraj Model and b) CMW Model  Health care providers and CMWs  2 years | **a)**  Intervention: 1095  Control: 1075  **b)**  Intervention: 712  Control: 1075 | Women (15 – 49) Years | Modern contraceptive prevalence rate  Contraceptive prevalence rate  Current use of method uses  Pill, injectable, condom, Female sterilization, Male Sterilization, IUD.  Knowledge of contraceptives methods  Pill, Condom, female sterilization, IUD, Injectable, Male Sterilization. | Age, education, province, number of children, and social-economic status | Moderate |
| Daniel,  2008,  India | Quasi-experimental  PRACHAR project  Female and male change agents  27 months | Intervention: 1447  Control: 633 | Women (15 – 24) Years | Contraceptive prevalence rate |  | Moderate |
| Dayal,  2003,  India | Quasi-experimental  Better Life Options Program  Better Life Option program alumnae.  3 years | Intervention: 835  Control: 858 | Women (15 – 26) Years | Contraceptive prevalence rate |  | Moderate |
| Douthwaite,  2005,  Pakistan | Quasi-experimental  Lady Health worker program  Lady health worker  6 years | Intervention: 3346  Control: 931 | Women (15 – 49) Years | Modern contraceptive prevalence rate  Contraceptive prevalence rate  Current use of method uses  Pill, injectable, condom, Female sterilization, IUD | Age, education, poverty, media, income | Moderate |
| Harries-Fry,  2016,  Bangladesh | Quasi-experimental  Participatory learning and action’ (PLA)  Facilitators  13 Month | Intervention: 2442  Control: 2686 | Women (15 – 49) Years | Modern contraceptive prevalence rate  Unmet Need | Baseline survey points, clustering | High |
| Hennink,  2005,  Pakistan | Quasi-experimental  Franchised Family planning clinics Health care providers  18-Month | Intervention: 1562  Control: 300 | Women (15 – 45) Years | Modern contraceptive prevalence rate  Pill, injectable, condom, Female sterilization, Male Sterilization, IUD  Knowledge of contraceptives methods  Pill, female sterilization, IUD, Injectable, Male sterilization | Baseline survey points, clustering | Moderate |
| Huda,  2019,  Bangladesh. | Quasi-experimental  Married Adolescent girls club (MAG club)  Facilitators  24 Month | Intervention: 749  Control: 2686 | Adolescent girls (14 – 19) Years | Modern contraceptive prevalence rate  Current use of method uses  Pill, injectable, condom, IUD, implants.  Knowledge of contraceptives methods  Pill, Condom, female sterilization, IUD, Injectable, Male sterilization, Implant  Emergency contraceptive use  Unmet Need | Education, employment status, age of marriage, number of pregnancies that the participant has experienced (including current pregnancy), background characteristics | High |
| Jeejeebhoy,  2015,  India | Quasi-experimental  Promoting Change in Reproductive Behavior in Bihar (PRACHAR) project  female and male change agents  27 Month | Intervention: 2130  Control: 716 | Women (15 – 34) Years | Modern contraceptive prevalence rate  Contraceptive prevalence rate  Knowledge of contraceptives methods  Pill  Condom  female sterilization  IUD  Injectable  Male sterilization  Emergency contraceptive use | Age, women’s and husbands’ educational attainment, women’s work status, caste, religion, household wealth, duration of residence in the study village, and number of surviving children | Moderate |
| Khan,  2008,  India | Quasi-experimental  Healthy Timing and Spacing of Pregnancy (HTSP) program  Community Workers | Intervention: 570  Control: 560 | Mean age of women 19.3 | Contraceptive prevalence rate  Current use by contraceptives methods  Pill, Condom, IUD  Knowledge by contraceptives methods  Pill, Condom  Emergency contraceptive use |  | Moderate |
| Kincaid,  2000,  Bangladesh | Quasi-experimental  Social network approach Family welfare Assistants (FWA) | Intervention: 107  Control: 2686 | Women (19 – 49) Years | Modern contraceptive prevalence rate | Parity, education, current contraceptive use, land ownership | High |
| Raj,  2016,  India | Randomized Control Trial  Counseling Husbands to Achieve Reproductive health and Marital equity(CHARM)  Village Male health care providers  18 Months | Intervention: 469  Control: 612 | Women (18 – 30) Years | Modern contraceptive Prevalence rate | Women’s age and education (any or none), caste or tribe, number of living sons, and number of living daughters, pregnancy intent | Moderate |
| Sebastian,  2012,  India | Quasi-experimental  Education provided by community health workers at homes and using materials (Booklet, poster, wall painting)  Auxiliary nurse midwives, accredited social health activists and community workers  1 Year | Intervention: 477  Control: 482 | Women (15 – 24) Years | Modern contraceptive Prevalence rate  Contraceptive prevalence rate  Current use of method use  Pill, condom, female sterilization, IUD  Knowledge by methods  Pill, condom, IUD  Current use of modern contraceptive by method | Education, age, cohabitation age, caste, parity | Moderate |
| Sood,  2004,  Nepal. | Quasi-experimental  Mass Media (Radio and Listening groups)  Female community health volunteers  1 Year | Radio Program and Listening Program  : 204  Only Radio Program  : 73  Control: 131 | Mean age of 33 years | Modern contraceptive Prevalence rate  Current use of method uses  Pill, injectable, condom, Female sterilization, IUD, Implant  Knowledge of modern contraceptives by methods  Pill, Condom, female sterilization, Injectable, Male sterilization, implants | Respondents’ age, sex education, caste, occupation, number of living sons | Moderate |
| Varkey,  2004,  India | Quasi-experimental  Facility based education for men and women individually or as a couple at antenatal clinics   Male and female doctors, laboratory technicians, Auxiliary Nurse midwives,  2 years | Intervention: 289  Control: 269 | Women (13 – 49) Years | Modern contraceptive rate  Contraceptive prevalence rate  Current use of method uses  Pill, Condom, female sterilization, IUD |  | High |
| Saifuddin Ahmed 2015 | Integrating FP Counseling with ongoing MNCH activities, delivered through trained staff and community health owrkers, 36 weeks | Intervention: 2247  Control: 2257 | All women of Reproductive age (14-49 years) | LAM, oral contraceptives, condoms, injectables | Age,  parity, socioeconomic status, women’s education, husband’s education, religion, fertility desire, and previous contraceptive use before the index pregnancy. | HIgh |
| Sarah Huber-Krum 2019 | stepped-wedge randomized controlled trial  Female community health volunteers and hospital staff  Two years | Intervention: 19298  Control: 19248 | Women of reproductive age | Modern contraceptive use, IUDC, | - | Moderate |
| Leon 2011 | Non-randomized experiment  Theater people trained  Health care providers  Intervention duration was two years | Intervention: 76000  Control: 77000 | Women of reproductive age (15-49 years) | Contraceptive use | Age, children, education, access to print and electricmedia | Moderate |
| Farid Midhet 2010 | Community Randomized control trail  Female volunteers trained at IEEC (information and education for empowerment and change | Intervention: 1539  Control: 1022 | Married women of reproductive age, Couples | Contraceptive use, maternal and neonatal health indicators | -- | High |

**APPENDIX D: Risk of Bias**

## Figure 1.1: Risk of Bias assessment for Quasi Experimental studies

|  | **Studies** | **Confounding** | **Selection** | **Classification of interventions** | **Deviations from intended interventions** | **Missing data** | **Measurement of outcomes** | **Reported result** | **Study Level Rob Judgement** |
| --- | --- | --- | --- | --- | --- | --- | --- | --- | --- |
| 1 | Ali 2019 |  |  |  |  |  |  |  |  |
| 2 | Ali 2020 |  |  |  |  |  |  |  |  |
| 3 | Azmat 2013 |  |  |  |  |  |  |  |  |
| 4 | Azmat 2016 |  |  |  |  |  |  |  |  |
| 5 | Dayal 2002 |  |  |  |  |  |  |  |  |
| 6 | Dowtwight 2005 |  |  |  |  |  |  |  |  |
| 7 | Harris 2016 |  |  |  |  |  |  |  |  |
| 8 | Hennick 2005 |  |  |  |  |  |  |  |  |
| 9 | Huda 2019 |  |  |  |  |  |  |  |  |
| 10 | Jejeboy 2015 |  |  |  |  |  |  |  |  |
| 11 | Kincaid 2000 |  |  |  |  |  |  |  |  |
| 12 | Sabistain 2012 |  |  |  |  |  |  |  |  |
| 13 | Sood 2004 |  |  |  |  |  |  |  |  |
| 14 | Denial 2015 |  |  |  |  |  |  |  |  |
| 15 | Khan 2008 |  |  |  |  |  |  |  |  |
| 16 | Varkey 2010 |  |  |  |  |  |  |  |  |
| 17 | Saifuddin Ahmed 2015 |  |  |  |  |  |  |  |  |
| 18 | Sarah Huber-Krum 2019 |  |  |  |  |  |  |  |  |
| 19 | Leon 2011 |  |  |  |  |  |  |  |  |

No Information

Serious

Moderate

Low

## Figure 1.2: Summary of Risk of Bias assessment for Quasi Experimental studies

## Figure 2: Risk of Bias assessment for Randomized Control Trials

|  |  | **D1** | **D2** | **D3** | **D4** | **D5** |  |
| --- | --- | --- | --- | --- | --- | --- | --- |
| Study | Intervention | Randomization process | Deviations from the intended interventions | Missing outcome data | Measurement of the outcome | Selection of the reported result | Overall assessment |
| Raj 2016 | CHARM |  |  |  |  |  |  |
| Farid Midhet 2010 | IEEC |  |  |  |  |  |  |

#

# **Appendix E: Sensitivity Analysis**

**The Sensitivity analysis included studies at low risk of bias for Incomplete data and confounding domain**

|  | **Odds ratio**  (Adjusted) | **95% Confidence Interval** | **P-value** |
| --- | --- | --- | --- |
| Primary Analysis 1.51 (1.35, 1.70) <0.00001 | | | |
| **Sensitivity Analysis** | | | |
| Incomplete data | 1.52 | (1.34, 1.73) | <0.00001 |
| Confounding | 1.51 | (1.30, 1.74) | <0.00001 |
| **Overall sensitivity analysis** | 1.50 | (1.28, 1.77) | <0.00001 |

# **Appendix F: Grade**

| **Certainty assessment** | | | | | | | **№ of patients** | | **Effect** | | **Certainty** | **Importance** |
| --- | --- | --- | --- | --- | --- | --- | --- | --- | --- | --- | --- | --- |
| **№ of studies** | **Study design** | **Risk of bias** | **Inconsistency** | **Indirectness** | **Imprecision** | **Other considerations** | **Outcomes** | **placebo** | **Relative (95% CI)** | **Absolute (95% CI)** |  |  |
| **Modern Contraceptive Prevalence Rate for Women of Reproductive Age (14-49)** | | | | | | | | | | | | |
| 15 | Randomized trials* | very^a^ serious | serious^b^ | not serious | serious^c^ | none | 8343/243990 (39.7%) | 17836/27263 (73.5%) | **OR 1.51** (1.35 to 1.70) | **75 more per 1,000** (from 55 more to 95 more) | ⨁◯◯◯ Very low | IMPORTANT |

*CI: confidence interval; OR: odds ratio *two cluster randomized trials and thirteen quasi-experimental*

Explanations

*a. Downgrade by 2 level: 1) The quasi nature of design has limitation of no randomization and allocation concealment 2) High risk of bias due to confounding*

*b. Downgraded by 1 level: Heterogeneity exists because the p value is 0.00001.*

*c. Downgraded by 1 level: The denominators have different no. in comparison groups*
